# Dissecting Abscesses of the Leg

**Published:** 1866-02

**Authors:** 


DISSECTING ABSCESS OF THE LEG. OPERATION; NITROUS-OXIDE GAS.—James ———, colored, æt. 15. Received a blow upon the left leg five weeks ago. An abscess has formed, which is about the middle of the limb on the outer side, immediately under the skin. The pus has worked its way in various directions, and has reached the surface by one small orifice at the inner side of the tibia. All this requires is a free incision, laying open the undermined portion. A flax-seed poultice and then some stimulating wash may be applied. Profound anæsthesia was induced by the nitrous-oxide, lasting one minute, and the abscess was freely opened. No unpleasant symptoms followed the use of the gas. Consciousness returned very rapidly. No sickness of the stomach.

